# Unilateral Parotid Abscess Within a Warthin’s Tumour Secondary to Brucellosis: A Case Report

**DOI:** 10.7759/cureus.117672

**Published:** 2026-10-09

**Authors:** Mohammad Alkraidees, Fareed Alghamdi, Khalid S Al Qarni, Mohammed A Alsaif, Ghusun Al Harbi

**Affiliations:** 1 Otolaryngology - Head and Neck Surgery, Prince Sultan Military Medical City, Riyadh, SAU; 2 College of Medicine, King Saud bin Abdulaziz University for Health Sciences, Riyadh, SAU; 3 College of Medicine, Qassim University, Unaizah, SAU

**Keywords:** abscess, brucellosis, parotid gland, parotid neoplasm, warthin's tumour

## Abstract

Brucellosis is a zoonotic multisystem disease that rarely causes infections in the head and neck region. *Brucella* infections of the parotid gland can lead to the formation of a parotid abscess, which can be associated with local and systemic complications.

We report a case of parotid gland abscess with associated Warthin’s tumour that presented acutely with pain, odynophagia and swelling. Imaging of the neck showed a parotid abscess with lymphadenopathy. Brucellosis caused by *Brucella melitensis* was confirmed on aspiration from the abscess. The patient required abscess drainage and a prolonged course of antibiotics, and significant clinical improvement was noted on follow-up, with resolution of the presenting symptoms.

In endemic countries, brucellosis should be considered as a differential diagnosis for unilateral parotid abscesses, especially in patients with occupational and health-related risk factors, and should be adequately investigated and treated to rule out other systemic complications.

## Introduction

Acute parotitis, characterized by swelling and inflammation of the parotid gland and reduced salivary flow, has a multifactorial aetiology, with viral and bacterial infections accounting for the majority of cases [1]. Parotitis can be complicated by abscess formation within or around the gland, either due to direct extension of the infection or due to involvement of intra-/periparotid lymph nodes via haematogenous spread [2]. A parotid abscess can be difficult to treat and can cause further complications, such as systemic infection or facial nerve involvement, and usually requires imaging and aspiration for diagnosis, with drainage of the abscess being the main treatment.

Brucellosis is a zoonotic multisystem disease caused by *Brucella* species, which have a seroprevalence of around 20% in Saudi Arabia [3]. The incidence of *Brucella* infections of the head and neck region is sparsely reported in the literature [4,5]. *Brucella* infections of the parotid gland are even rarer, with only two reported cases associated with Sjögren disease and Warthin’s tumour [6,7].

In this report, we present the case of a unilateral parotid abscess caused by *Brucella melitensis* within a Warthin’s tumour in a patient with animal exposure and immunocompromise secondary to diabetes and chronic smoking, which is the fourth such case described in the literature, to our knowledge.

## Case presentation

A 46-year-old male was admitted through the Emergency Department with a painful mass on the right side of his neck that had been increasing in size over the past month and was associated with painful swallowing. The patient was noted to be febrile and to have a cough, but had no symptoms of respiratory distress, stridor, recent neck trauma, or other head and neck lesions. His past medical history was positive for type 2 diabetes, psychiatric disorder, and smoking (30 pack-years). He had a history of animal contact with camels. Based on the presentation, he was referred to the otorhinolaryngology team for further assessment.

On physical examination, he was hemodynamically stable but febrile, with a temperature of 38.5°C. Examination of the oral cavity showed poor dental hygiene, and he appeared dehydrated. He was noted to have an erythematous and hard mass around 4 × 4 cm in size over the right parotid gland, extending posteriorly to the sternocleidomastoid muscle. The mass was not fluctuant, and no purulent discharge could be expressed from the right Stensen's duct on bimanual massage of the gland. He did not have any trismus, restriction of tongue movements, or cervical lymphadenopathy. His flexible nasal endoscopic examination was also unremarkable.

Blood investigations, as shown in Table 1, indicated poor diabetic control, with glycated haemoglobin of 12.21%. He was also noted to have raised inflammatory markers, with a C-reactive protein (CRP) level of 107.5 mg/L and a leucocyte count of 9.1 × 10⁹/L, with predominant neutrophilia. His renal and liver function were within normal limits, and he had mild electrolyte derangements, including hypokalaemia, hypocalcaemia, and hyperphosphataemia. His thyroid profile and haematinics were normal. The patient was started on antipyretics and intravenous antibiotics (ceftriaxone and clindamycin), with close glucose monitoring and management. Despite symptomatic improvement over 24 hours, his swelling persisted, and a computed tomography (CT) scan of the head and neck was performed. This revealed a multiloculated, heterogeneous, peripherally enhancing right parotid lesion with adjacent inflammatory changes and upper cervical lymphadenopathy, suggestive of abscess formation (Figure 1).

**Table 1:** Selected laboratory results on admission. ↑ = above reference range; ↓ = below reference range. Reference ranges are provided as standard adult values; institutional laboratory reference intervals may differ slightly. CRP, C-reactive protein; HbA1c, glycated haemoglobin; HIV, human immunodeficiency virus; WBC, white blood cell; AST, aspartate aminotransferase; ALP, alkaline phosphatase; ALT, alanine aminotransferase; TSH, thyroid-stimulating hormone; eGFR, estimated glomerular filtration rate

Laboratory Parameter	Patient Value	Reference Range	Interpretation
Glycaemic Control			
HbA1c	12.21%	<5.7%	↑ (Poor control)
Inflammatory Markers			
CRP	107.5 mg/L	<5.0 mg/L	↑ (Elevated)
WBC count with predominant neutrophilia	9.1 × 10⁹/L	4.0-11.0 ×10⁹/L	Normal (neutrophilia)
Electrolytes			
Potassium (K⁺) - mild hypokalaemia	Low (mild)	3.5-5.0 mmol/L	↓ (Mild)
Calcium (Ca²⁺) - mild hypocalcaemia	Low (mild)	2.1-2.6 mmol/L	↓ (Mild)
Phosphate (PO₄³⁻) - mild hyperphosphataemia	High (mild)	0.8-1.5 mmol/L	↑ (Mild)
Serology			
HIV serology	Negative	Negative	Normal
Syphilis serology	Negative	Negative	Normal
*Brucella melitensis* antibody titre	1:640 (positive)	<1:160 (negative)	↑ (Positive)
Renal and Liver Function			
Renal function tests (urea, creatinine, eGFR)	Within normal limits	Laboratory-specific	Normal
Liver function tests (AST, ALT, ALP, bilirubin)	Within normal limits	Laboratory-specific	Normal
Thyroid profile (TSH, free T4)	Within normal limits	Laboratory-specific	Normal
Haematinics (iron, folate, vitamin B12)	Within normal limits	Laboratory-specific	Normal

**Figure 1:**
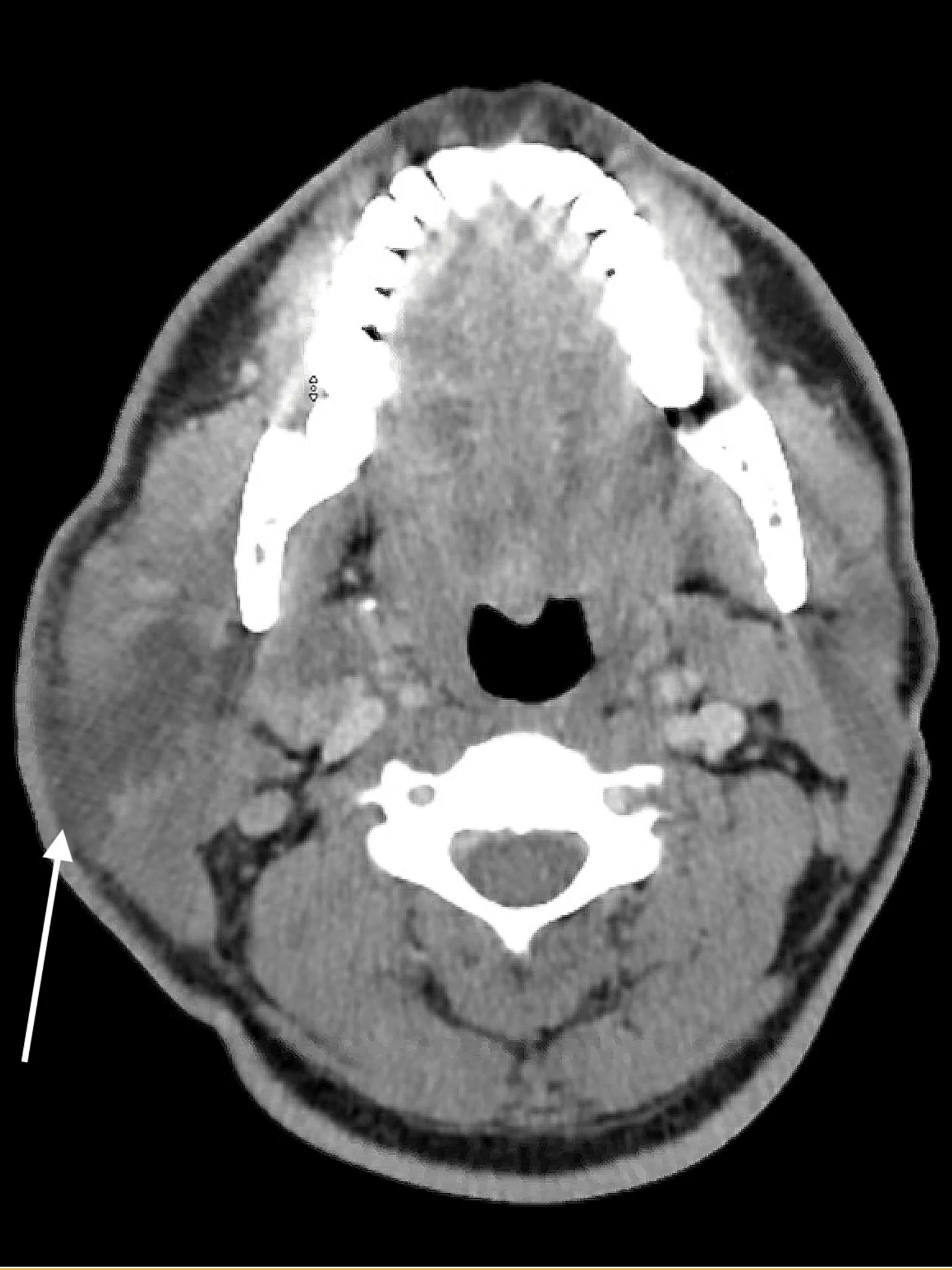
Multiloculated, heterogeneous, peripherally enhancing lesion within the right parotid gland, with extension into the deep lobe, measuring 5.5 × 4.5 × 2.1 cm (arrows; transverse, anteroposterior, and craniocaudal dimensions).

Blood investigations showed negative serologies for HIV and syphilis; however, his *B. melitensis* antibody titre was positive at 1:640. The patient underwent needle aspiration of the abscess, as well as a core biopsy of the right parotid gland. Culture of the pus obtained by needle aspiration grew *B. melitensis*, with microscopy revealing multiple polymorphonuclear neutrophils and debris in the background. A needle core biopsy of the right parotid gland was obtained concurrently. The specimen comprised multiple fragments of cream-tan soft tissue measuring 0.3 × 0.4 cm in aggregate, submitted in a single cassette, and was reported as Warthin's tumour of the right parotid gland.

The patient was started on intravenous gentamicin and doxycycline based on the sensitivity results from the specimen. He also underwent repeat ultrasound-guided drainage of the parotid gland, with further pus drained, leading to marked symptomatic improvement. A repeat CT scan of the neck with contrast, performed four days after the initial aspiration, demonstrated a multiloculated collection in the right parotid region. Rather than representing a residual collection, this finding indicated interval enlargement. Several factors contributed to this finding, including internal air foci, which reflected air introduced during aspiration, and reaccumulation of the collection, which was consistent with ongoing infection (Figure 2). The patient received a 14-day course of antibiotics (gentamicin and doxycycline) and analgesia and was discharged from hospital two weeks after the initial presentation.

**Figure 2:**
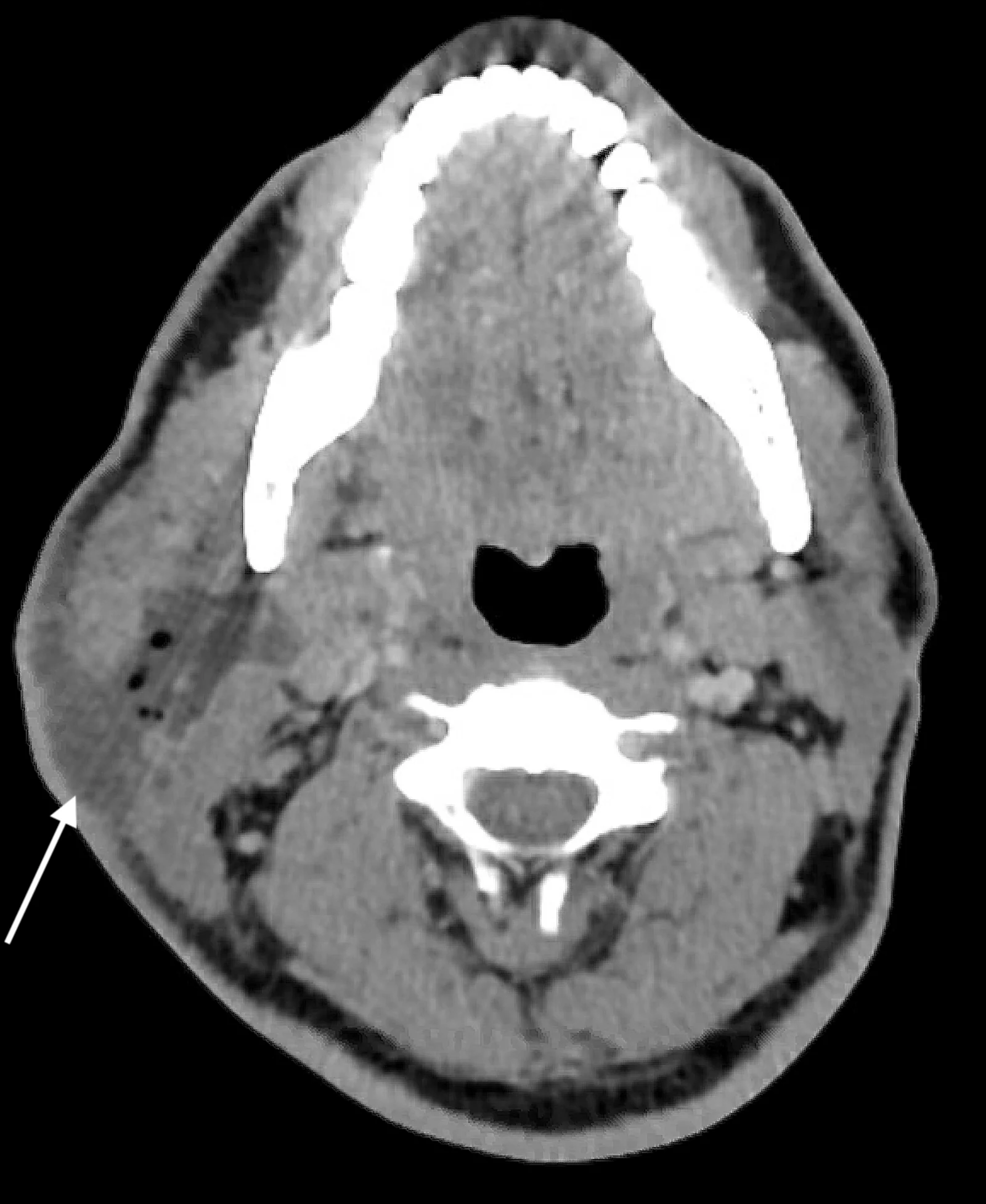
Fluid collection in the right parotid region involving the superficial and deep lobes, measuring 5.2 × 5.3 × 6.5 cm, with surrounding fat stranding and internal air foci (arrows).

Follow-up at six months with the infectious diseases clinic and the otorhinolaryngology team showed no complications, continued regression of the parotid mass, and good symptomatic control.

## Discussion

Acute suppurative parotitis, usually of infectious aetiology, can often lead to unilateral severe pain with redness and swelling in the parotid area and can result in localized pus collection with glandular enlargement and inflammatory changes [8]. Predisposing factors for parotid abscesses include systemic conditions such as diabetes mellitus, rheumatological conditions such as Sjogren disease, immunocompromised state, and significant dehydration [7]. Parotid abscesses can also potentially spread to deep neck spaces, leading to systemic infections and significant complications such as mediastinitis, upper airway obstruction, jugular vein obstruction, and septic shock [9]. While incision and drainage of abscesses is an appealing treatment option, evidence from a recent systematic review recommends antibiotic therapy as the mainstay of treatment, with drainage reserved as a salvage option for conservative treatment failure [10].

Warthin’s tumour is the second most common benign neoplasm of the salivary glands and accounts for up to 15% of all primary epithelial tumours of the parotid gland. It is male-predominant and more common in smokers, with other risk factors being Epstein-Barr virus infection, autoimmune diseases, and chronic inflammation [11]. Patients with Warthin’s tumour can be predisposed to parotid abscess formation due to multiple factors, such as the cystoid nature of the tumour, with a fluid-filled cavity acting as a milieu for abscesses, as well as lymphoid tropism and compression by the neoplasm, leading to stasis and an increased risk of infection. Other contributing factors to abscess formation include ductal ectasis, primary parenchymal involvement, or infection of periparotid or intraparotid lymph nodes; systemic conditions such as diabetes can also increase the likelihood of abscess formation due to microvascular compromise [6].

Brucellosis is a multisystem infection spread via both direct and indirect animal contact, predominantly via unpasteurized dairy products. Other modes of spread can include inhalational transmission or transmission via mucous membranes. Systemic infections with *Brucella* present with malaise, headache, anorexia, joint pains, splenomegaly, and abdominal tenderness [12]. *Brucella* infection can also lead to abscess formation in various regions of the body, although reporting of head and neck abscesses is quite rare and usually occurs in endemic regions [13]. Warthin's tumour is a benign salivary gland neoplasm with no malignant potential in the overwhelming majority of cases. Its relevance to abscess formation is structural rather than oncological. *Brucella* infection has separately been described in patients with underlying malignancy [14].

The patient in our case had chronic smoking as a risk factor for Warthin’s tumour, and his poor dental hygiene and uncontrolled diabetes also contributed to *Brucella* infection with parotid abscess in an endemic area with animal contact. While serological tests such as tube agglutination, Rose Bengal staining, and enzyme-linked immunosorbent assay (ELISA) can help in the diagnosis of *Brucella*, the isolation of *Brucella* species in culture fluid from infected tissues is more specific, and sensitivity results should be used to guide further treatment [7,15].

Imaging is important in acute parotitis to confirm glandular inflammation and to rule out stones, abscesses, or tumours [1]. However, in cases of *Brucella* infections of the head and neck region, imaging can be essential to rule out significant local spread or systemic complications. Long-term *Brucella* infections can lead to imaging features that mimic other chronic granulomatous diseases, such as tuberculosis or malignancy, and therefore, imaging should be supplemented with diagnosis from abscess aspiration or with serological testing [4]. In head and neck infections, *Brucella* can also infect the nervous system, leading to neurobrucellosis with manifestations such as meningitis or multiple cranial nerve involvement [16,17]. These were excluded in our case, with a normal lumbar puncture.

Treatment for brucellosis includes antibiotic therapy such as tetracyclines, trimethoprim/sulfamethoxazole, third-generation cephalosporins, and rifampin [15]. Several weeks of treatment are usually required as the bacteria reside within cells; furthermore, combination antimicrobial therapy is usually preferred over a single antibiotic due to the high risk of relapse. There is a limited role for corticosteroids in *B. meningitis* and in severe cases of brucellosis. Surgical treatment with abscess drainage can be indicated in patients with a slow or poor response to antimicrobial treatment. Patients need regular monitoring during treatment to ensure disease control, as complications and relapses can occur; patient education is also particularly important regarding avoidance of infected animals and contaminated food, pasteurization of dairy products, and immunization of cattle, sheep, or camels [18,19]. Control of risk factors, such as tight glycaemic control and quitting smoking, can also be helpful in preventing relapses and systemic complications. 

The three previously reported cases differ from ours in instructive ways. Horasan et al. described *B. melitensis* infection within a Warthin tumour, but their patient presented with an enlarging preauricular mass rather than with acute infective features and was managed by superficial parotidectomy combined with medical therapy [6]. Yolbaş et al. reported parotid abscess in a patient with primary Sjögren's syndrome, in whom glandular hypofunction and reduced salivary flow provided the predisposing environment [7]. Elkaruori and Mohamed described *Brucella* parotitis with abscess formation whose course was complicated by a paradoxical reaction during therapy [19]. Our patient is distinguished by an acute suppurative presentation with fever, pain, and odynophagia, and by successful management without parotidectomy, using ultrasound-guided aspiration and repeat drainage alone. Taken together, these cases suggest that the predisposing lesion determines the presentation, while the completeness of source control determines the treatment duration required.

One limitation should be acknowledged. The lesion was not excised in its entirety, and the diagnosis of Warthin's tumour rests on a needle core biopsy rather than on microscopic examination of a resected specimen. Combined with the multiloculated nature of the collection and its involvement of both parotid lobes, this means we cannot demonstrate that the abscess arose within the tumour itself rather than in adjacent parenchyma or intraparotid lymph nodes containing a coincident Warthin's tumour. An intratumoural origin remains the most plausible explanation on anatomical and mechanistic grounds, but it is an inference, not a histologically proven finding.

## Conclusions

Our case presents a rare manifestation of brucellosis in a parotid abscess arising in association with a Warthin’s tumour, which, to our knowledge, has only been reported three times before, particularly in the absence of systemic symptoms. Brucellosis should be considered in the differential diagnosis of parotid abscesses, especially in endemic areas, and careful examination should be performed to exclude secondary abscesses and systemic symptoms.
